# Supplementary material for: Causal Relationship between Aging and Anorexia Nervosa: A White-Matter-Microstructure-Mediated Mendelian Randomization Analysis
Source: Biomedicines. 2024 Aug 16;12(8):1874. doi: 10.3390/biomedicines12081874 (PMC11351342; doi:10.3390/biomedicines12081874)
Supplement: Supplementary file 1 [file biomedicines-12-01874-s001.zip › Supplementary Figure/Figure S9/Leaveoneout plot of MR analysis for ubm-b-1845 to AN.pdf]

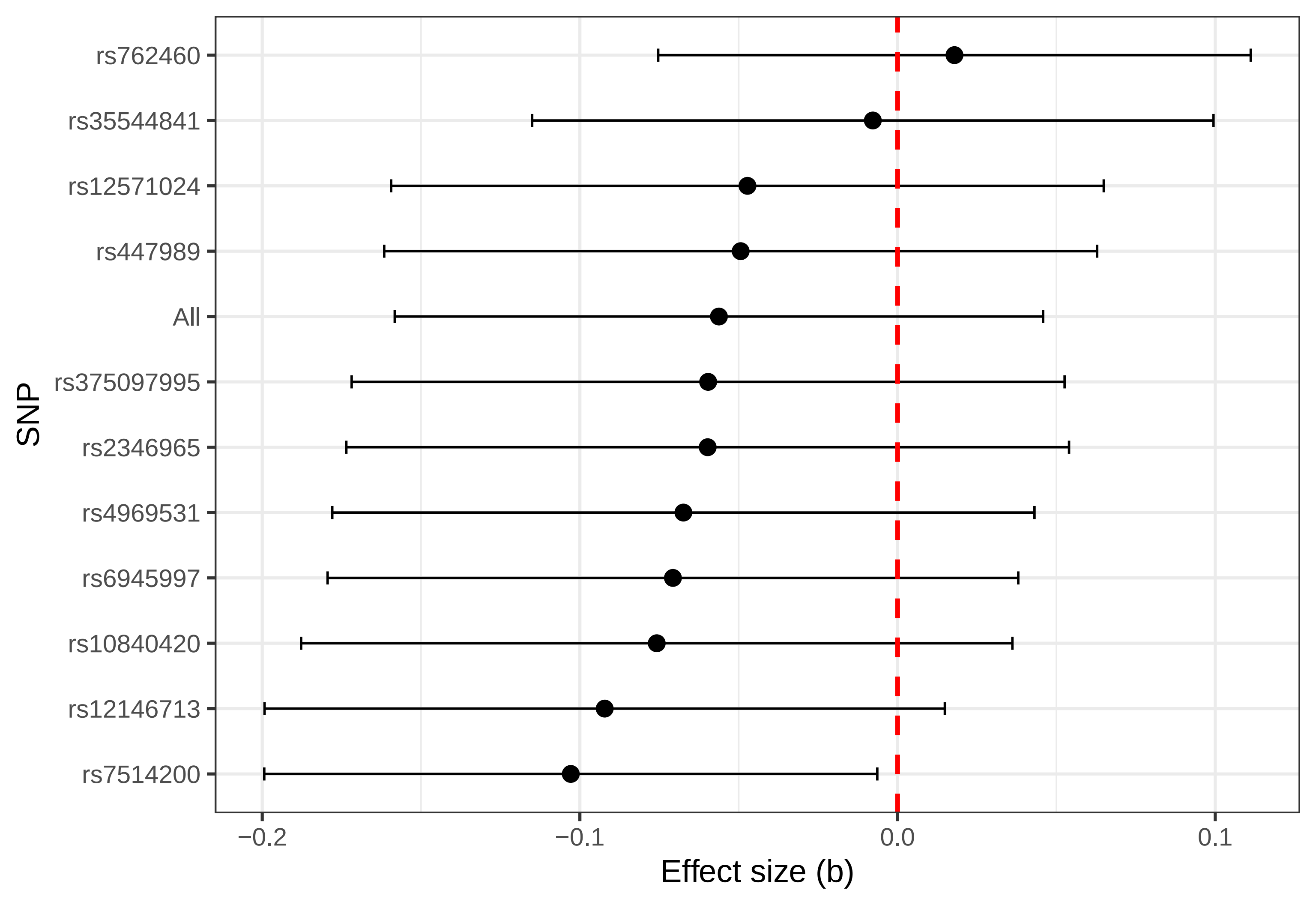
rs762460
rs35544841
rs12571024
rs447989
All
rs375097995
rs2346965
rs4969531
rs6945997
rs10840420
rs12146713
rs7514200
SNP
−0.2
−0.1
0.0
0.1
Effect size (b)
